# Coronavirus Disease-Associated Pulmonary Aspergillosis: A Devastating Complication of COVID-19

**DOI:** 10.7759/cureus.13004

**Published:** 2021-01-30

**Authors:** Adeel Nasrullah, Anam Javed, Khalid Malik

**Affiliations:** 1 Internal Medicine, Allegheny Health Network, Pittsburgh, USA; 2 Pulmonary and Critical Care Medicine, Allegheny Health Network, Pittsburgh, USA

**Keywords:** corona virus disease 2019 (covid-19), invasive aspergillosis, vv-ecmo, immuno-competent

## Abstract

Coronavirus disease-19 (COVID-19) has affected more than ninety-three million people worldwide till January 2021. COVID-19 can cause a destructive dysregulated immune response which can result in numerous complications such as kidney failure, myocarditis, and strokes. A new entity called coronavirus disease-associated pulmonary aspergillosis (CAPA) has emerged in recent times. The literature on CAPA is limited. We present a case of CAPA in an immunocompetent patient who was placed on veno-venous extra-corporeal membranous oxygen (VV-ECMO). We briefly explained pathophysiology, clinical presentations, and management of CAPA in this report.

## Introduction

Coronavirus disease-19 (COVID-19) can cause extensive damage to airway epithelial cells due to exaggerated inflammation. The latter impedes virus clearance and causes secondary infections. Handful cases of coronavirus disease-associated pulmonary aspergillosis (CAPA) have been reported so far, mostly in Europe and China [1]. CAPA confers a very poor prognosis and has varied radiologic presentations, unlike classic angio-invasive aspergillosis, therefore a high suspicion is required to investigate and appropriately treat CAPA. We present a case of a fairly healthy gentleman who had a prolonged intensive care unit (ICU) stay due to the need for mechanical ventilation and veno-venous extra-corporeal membranous oxygen (VV-ECMO) for respiratory failure secondary to COVID-19 and superimposed bacterial pneumonia followed by CAPA. 

## Case presentation

A 68-year-old man with a history of type 2 diabetes mellitus and hypertension was admitted to the hospital with complaints of persistent fever, cough, and lethargy for one week. On arrival to the hospital, his vitals were blood pressure 130/77 mm of Hg, heart rate 102 beats per minute, respiratory rate 28 per min, oxygen saturation 87% on room air requiring 3 L oxygen via nasal cannula to maintain oxygen saturation greater than 95%. The patient tested positive for COVID-19 through reverse transcriptase-polymerase chain reaction (PCR) testing. He received a unit of convalescent plasma, remdesivir 200 mg on day one followed by 100 mg daily for five days, and dexamethasone 6 mg daily for ten days. He continued to have persistent severe hypoxia refractory to non-invasive ventilation and self-proning ultimately requiring mechanical ventilation on day ten of hospitalization. Despite lung-protective ventilation, proning, paralysis, and aggressive diuresis, the patient continued to have refractory hypoxia. A shared decision was made by the multidisciplinary team to institute VV-ECMO on the eighth day of mechanical ventilation. Hospital course was further complicated by septic shock secondary to pneumonia due to *Hemophilus influenza and Pseudomonas aeruginosa*, requiring broad-spectrum antibiotics, vasopressors, and stress dose steroids. The patient received steroids for nineteen days.

**Figure 1:**
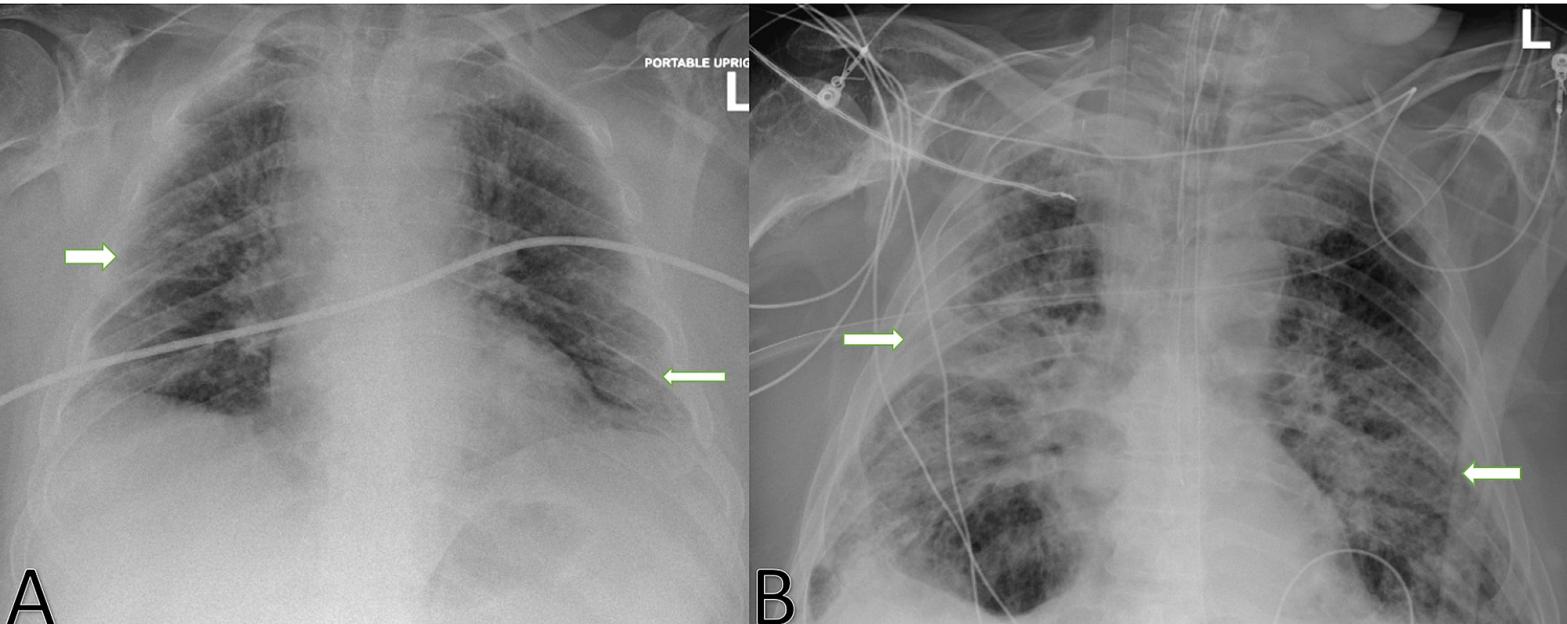
Posterior-anterior view of chest X-ray (CXR) at admission (A) and at the diagnosis of invasive aspergillosis (B) CXR on the left (A) shows bilateral patchy infiltrates as marked by white arrows, while CXR on the right (B) shows dense multilobar patchy consolidations marked by white arrows.

Despite aggressive treatment of ventilator-associated pneumonia, the patient continued having persistent lung infiltrates, refractory hypoxia, fever, and leukocytosis. Work-up for superimposed fungal infection with serum fungitell and galactomannan was negative. A bedside bronchoscopy with bronchoalveolar lavage (BAL) was performed for further evaluation and fungal studies were obtained through BAL. BAL *Aspergillus* galactomannan antigen resulted strongly positive with an index of 2.16 (0.00-0.49) and BAL cultures grew *Aspergillus fumigatus*. A comparison between chest X-ray at admission and at the time of diagnosis of invasive aspergillosis is shown in Figure 1.

**Figure 2:**
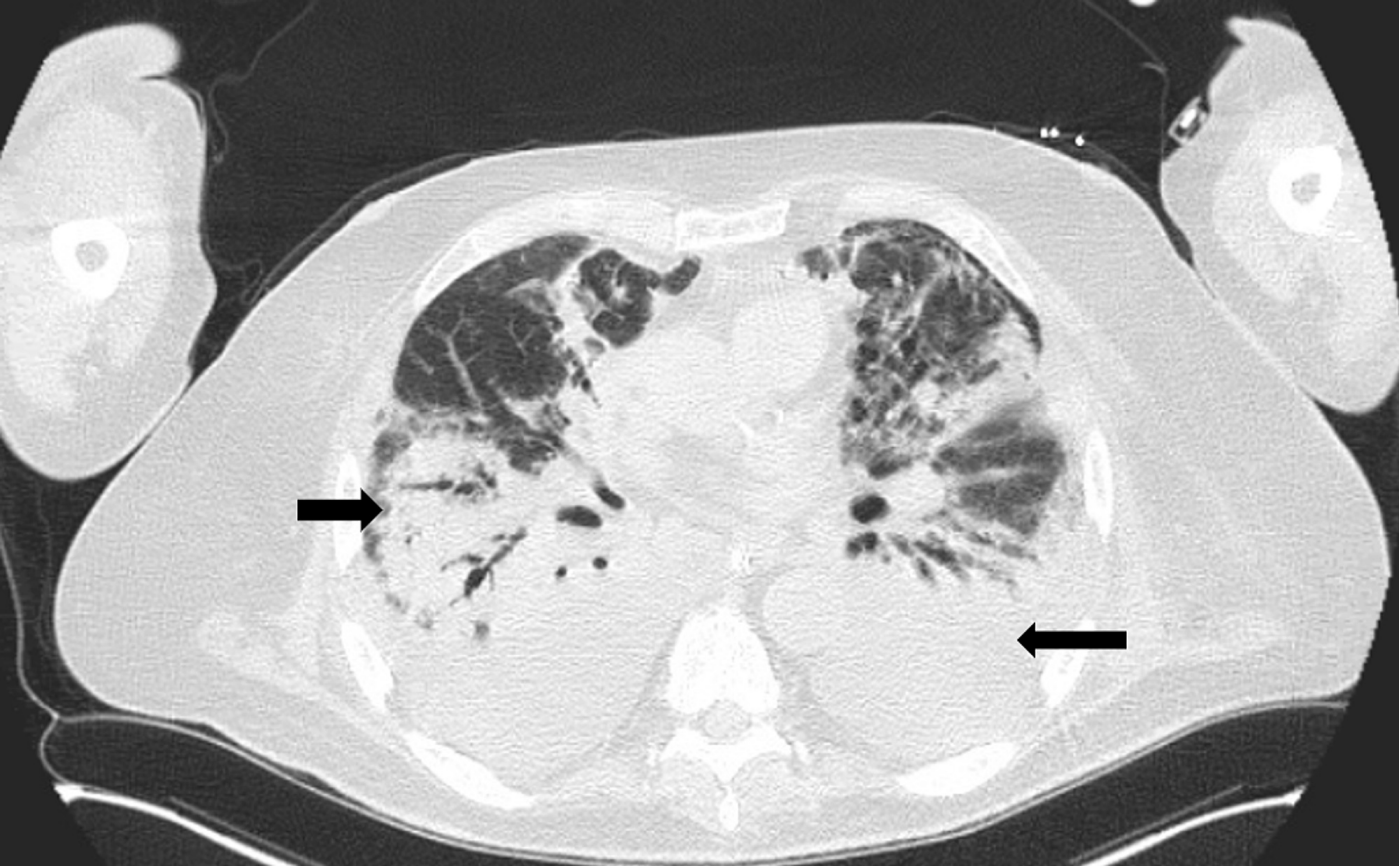
CT chest shows multilobar diffuse consolidations as marked by black arrows

Computed tomography (CT) scan of the chest showed patchy multilobar consolidations consistent with multilobar pneumonia and acute respiratory distress syndrome (ARDS) (Figure 2).

Patient symptoms have met the criteria of possible invasive Aspergillosis. He received seven days of voriconazole and a repeat second test for aspergillus galactomannan antigen from tracheal aspirate was negative. Citing a protracted disease course characterized by forty-two days of mechanical ventilation and thirty-four days on VV-ECMO without resultant improvement, the patient was transitioned to comfort care and passed away shortly thereafter.

## Discussion

Invasive aspergillosis is a known complication among patients with severe influenza pneumonia with a reported incidence of 19% [2]. Due to the COVID-19 pandemic, several cases of CAPA are reported in the literature, although it could be underreported due to a lack of high suspicion [3]. CAPA is associated with high all-cause mortality and its true incidence is still unknown due to the evolving understanding of the disease. In the literature, CAPA has been reported in 3.3%-4% of all COVID-19 admitted patients [4,5]. Though its incidence can be as high as 27.1% in patients with severe ARDS and elevated interleukin 6 (IL-6) levels [6]. Unlike invasive aspergillosis which typically affects immunocompromised patients, CAPA has been seen in immunocompetent patients who are suffering from severe ARDS requiring steroids [4]. Frequently reported risk factors include old age, chronic lung disease, uncontrolled diabetes, mechanical ventilation, prolong steroids, and antibiotics use [4,7]. COVID-19 typically results in an exuberant inflammatory response resulting in the sloughing of airway epithelial cells and poor clearance of viruses. This allows aspergillus conidial invasion of the airway leading to inflammation and necrosis in severe cases. Most CAPA patients do not meet the host factors criteria of the European Organization for Research and Treatment of Cancer and the Mycoses Study Group Education and Research Consortium (EORTC/MSGERC) for diagnosis of invasive aspergillosis, so a clinical algorithm as per AspICU study is used [8].

Diagnosis is established by obtaining cultures from trans-tracheal aspirates and aspergillus galactomannan index in patients with a high degree of clinical suspicion. CT chest shows various presentations but the majority of patients with COVID-19 have diffuse peripheral ground-glass opacities in severe disease. Superimposed CAPA in COVID-19 patients can have nodules with or without necrosis, a tree in bud appearance, or progressive peribronchial consolidations on chest CT. Due to airway invasive disease with CAPA, classic angio-invasive aspergillosis findings such as cavities, halos signs with nodule have not been frequently observed. This is also consistent with the fact that galactomannan was detected in 77% of BAL samples as compared to 42% in serum in one of the studies [4]. Studies have suggested mortality rates ranging from 44% to 100% [4,9]; the lack of reciprocity among results can be partly explained by aspergillosis colonization rather than active infection.

Therapeutic options include isavuconazole or voriconazole as the first line of treatments. Due to difficulties in monitoring voriconazole levels in COVID-19 patients, isavuconazole should be preferred. Definitive antifungal therapy must be tailored based on final culture results and antifungal sensitivities as resistance to azoles is not uncommon. Following the Randomised Evaluation of COVID-19 Therapy (RECOVERY) trial and COVID-19-associated ARDS treated with DEXamethasone (CoDEX) trial, steroids have been used extensively in the management of COVID-19, often for prolong courses than recommended (>10 days) due to the disease severity [10,11]. Such protracted courses can further predispose patients to secondary fungal infections and can also result in uncontrolled hyperglycemia which further imparts a poor prognosis in this patient population.

## Conclusions

CAPA is associated with significant morbidity and a high mortality rate. We suggest keeping a low threshold to investigate for CAPA in COVID-19 patients in an appropriate clinical setting as early detection and treatment may improve outcomes. Moreover, prolonged courses of steroids should not be given unless further conclusive evidence is available.
